# Methods for Using Small Non-Coding RNAs to Improve Recombinant Protein Expression in Mammalian Cells

**DOI:** 10.3390/genes9010025

**Published:** 2018-01-09

**Authors:** Sarah Inwood, Michael J. Betenbaugh, Joseph Shiloach

**Affiliations:** 1 Biotechnology Core Laboratory, NIDDK, NIH, Bethesda, MD 20892, USA; sarah.inwood@nih.gov; 2 Department of Chemical and Biomolecular Engineering, Johns Hopkins University, Baltimore, MD 21218, USA; beten@jhu.edu

**Keywords:** microRNA, screening, microarray, microRNA library, next generation sequencing

## Abstract

The ability to produce recombinant proteins by utilizing different “cell factories” revolutionized the biotherapeutic and pharmaceutical industry. Chinese hamster ovary (CHO) cells are the dominant industrial producer, especially for antibodies. Human embryonic kidney cells (HEK), while not being as widely used as CHO cells, are used where CHO cells are unable to meet the needs for expression, such as growth factors. Therefore, improving recombinant protein expression from mammalian cells is a priority, and continuing effort is being devoted to this topic. Non-coding RNAs are RNA segments that are not translated into a protein and often have a regulatory role. Since their discovery, major progress has been made towards understanding their functions. Non-coding RNA has been investigated extensively in relation to disease, especially cancer, and recently they have also been used as a method for engineering cells to improve their protein expression capability. In this review, we provide information about methods used to identify non-coding RNAs with the potential of improving recombinant protein expression in mammalian cell lines.

## 1. Introduction

The ability to produce recombinant proteins by utilizing different “cell factories” revolutionized the biotherapeutic and pharmaceutical industry, and consequently influenced health care operations worldwide [1]. Proteins can be produced in different prokaryotes and eukaryotes, such as bacteria, fungi, yeast, insect cells, and mammalian cells [2]. Mammalian cells are most suitable for pharmaceutical purposes because of their ability to biosynthesize complex proteins, and, therefore, are currently the preferred producers [3,4]. Chinese hamster ovary (CHO) cells are the dominant industrial producer, especially for antibodies, since they are able to grow in suspension in chemically defined media, are resistant to viral infection, and secrete high quality protein with some post-translational modifications that are similar to those of the human proteins [5]. Therefore, improving recombinant protein expression from CHO cells is a priority and continuing effort is being devoted to this since the first therapeutic protein, human tissue plasminogen activator, was approved [6]. Approaches such as improving metabolism, glycosylation, anti-apoptosis and pro-proliferation, molecular chaperones, and protein folding have been successfully implemented [7,8]. Human embryonic kidney cells (HEK), while not being as widely used as CHO cells, are used for purposes where CHO cells are unable to meet the needs for the expression of membrane proteins, specific growth factors, and isolated receptor channels [9]. Generally, since HEK cells are human cells, they are more suitable than non-human cell lines for producing recombinant human proteins with proper post-translation modifications that are associated with correct folding to produce a preferred product [10,11,12,13].

Small non-coding RNAs are primarily short RNA segments that are not translated into a protein. Since their discovery, a great deal of progress has been made towards understanding their function [14,15,16]. The microRNA is an example of a small regulatory non-coding RNA that is approximately 23 nucleotides long in mature form [17]. The sequence includes a seed region that can promiscuously bind to multiple mRNA molecules and most often represses them by initiating degradation or translation inhibition. Small interfering RNA (siRNA) and short hairpin RNA (shRNA) are also types of non-coding RNA molecules, among many others [18]. Non-coding RNA has been investigated extensively in relation to disease, especially cancer, and recently have also been used as a method for engineering cells to improve protein expression [19,20]. Several reviews have been published on the utilization of microRNA for optimizing protein expression from CHO cells. These reviews focused on using microRNAs to engineer process improvements, such as cell growth improvement and apoptosis reduction [20,21,22,23,24]. In this review, we provide a discussion of the methods that are used to identify non-coding RNAs with the potential of improving recombinant protein expression in mammalian cell lines.

## 2. MicroRNA Screening Tools

MicroRNAs are currently the most frequently used non-coding RNA for improving CHO and HEK cell protein production capabilities. MicroRNAs can target multiple genes in the same pathway, making them good targets of a specific cell process, such as reducing apoptosis, leading to improved protein production [22]. Initial work was done in 2007 by Gammell et al. [25] profiling microRNAs of CHO-K1 suspension cells during batch culture, at two different temperatures by using cross-species microRNA microarrays. Following this work, other investigator began researching the possibilities using microRNAs to improve protein expression and multiple microRNAs were evaluated for the expression of a range of recombinant proteins, as described in the following sections. A variety of screening methods were utilized for identifying specific microRNAs that can potentially improve the expression of proteins. These methods include using previously identified microRNAs, microarrays, microRNA screens, and next generation sequencing (NGS) (See Table 1). 

### 2.1. Utilization of Previously Identified microRNAs 

Several microRNAs that were previously identified to affect specific growth properties of mammalian cells were tested for their possible effects on improving the expression of recombinant proteins. For example, in 2015, Kelly et al. [26] made use of the knowledge that the mir-34 family has pro-apoptotic and anti-proliferative function. By transient transfection of mir-34 mimics and a stable mir-34 sponge, they tested the effect on expressing secreted alkaline phosphatase (SEAP) in CHO cells. These experiments showed that mir-34 had a negative effect on the SEAP productivity of the CHO cells, and microRNAs could be selected as targets for improving protein expression based on their functions.

Another study in 2015 [27] explored the effect of mir-23 on CHO cells producing SEAP based on the role of mir-23 in energy metabolism. CHO cells expressing SEAP, which were stably depleted of mir-23, demonstrated improved SEAP productivity at the transcriptional level. Further exploration looked at the mitochondrial function and proteomic analysis using LC-MS examined potential targets.

### 2.2. Microarrays Utilization

Microarrays are chips containing probes for the purpose of detecting differentially expressed microRNAs or mRNA in an RNA extract [44]. Microarrays made it possible to engineer cells that target microRNAs that are expressed in specific culture conditions, such as apoptosis or temperature shifts. Gammell et al. [25] were the first to explore the possibility of using human, mouse, and rat microRNA probes in the microarray format for analyzing CHO-K1 microRNA expression. They compared the microRNA profiles of suspension culture at two different temperatures, using human cell lines as a reference. A quantitative real-time polymerase chain reaction (qRT-PCR) was used to validate five selected microRNAs. Two microRNAs, hsa-mir-21 and hsa-mir-24, were confirmed as being differentially regulated between the two temperature conditions. The *Cricetulus griseus* cgr-miR-21 was then isolated and cloned. In 2011, Barron et al. [29] used Human TaqMan Array MicroRNA cards (TLDA) to detect microRNAs that were differentially expressed during temperature shift of CHO cells. By following this analysis with qRT-PCR and mir-mimic and anti-mir transfections, they were able to identify mir-7 as a target for increasing cell proliferation and improving productivity of secreted alkaline phosphatase (SEAP) from the CHO cells. Following the identification of mir-7 as a target, Meleady et al. [45] investigated its impact on the cell proteome by using LC-MS/MS. They found that ribosomal and histone proteins, which also regulate growth and proliferation, are significantly downregulated. Two genes in cell growth, *stmn1*, which encodes stathmin, and *cat*, which encodes catalase, were identified as possible direct targets of mir-7. The researchers later generated stable clones with a mir-7 sponge decoy that improved cell density, viability, and secreted protein in a fed batch culture [46].

In 2009, microarrays that were designed to probe human and mouse microRNAs were used to identify differentially expressed microRNAs in different growth stages of HEK 293 cells grown in a bioreactor [28]. By using this approach, Koh et al. were able to identify 13 microRNAs that were upregulated and one that was down-regulated in the exponential phase when compared with their expression in the stationary phase. These microRNAs were related to apoptosis, growth arrest, and differentiation. The researchers speculated that the identified microRNAs could be used to control cell cycle regulation, enhancing the cell growth of both HEK and CHO cells.

Another example of utilizing microarray for microRNAs identification is the library search that was conducted for microRNAs that induce apoptosis [30]. Apoptosis was induced in CHO cells by exposing the culture to nutrient depleted media and the microRNAs expression profile was evaluated by using microarrays with mouse and rat microRNAs. Following cluster analysis, mmu-mir-446-5p was selected for follow-up with qPCR and transient transfection with anti-mir. Bioinformatics was then used to identify targets for this microRNA and narrow the list to the following apoptosis related genes: *bcl2l2*, *dad1*, *birc6*, *stat5a*, and *smo*. Druz et al. [47] then examined the time-dependent activation of miR-466h-5p, miR-669c, and the *Sfmbt2* gene following glucose deprivation-induced oxidative stress which caused inhibition of histone deacetylation in mouse cells. Next, stable inhibition of mmu-mir-446h-5p by expression of anti-mir-446h-5p was done and the resulting engineered CHO cell line demonstrated improved apoptosis resistance together with the enhanced production of SEAP [48].

In 2011, a microarray analysis of human, mouse, and rat microRNAs was used successfully to compare the microRNA profile of two CHO cell lines producing IgG with parental DG44 cell line [31]. After selecting 16 microRNAs, Lin et al. [31] proceeded to validation with qRT-PCR of four IgG-producing lines with varying degrees of productivity. Following the qRT-PCR analysis of the effect of amplification with Methotrexate on the microRNA was explored as well as a comparison to CHO K1. Bioinformatics analysis was performed to identify predicted targets of the five selected differentially expressed microRNAs, mir-221, mir-222, mir-19a, let-7b, and mir-17. Target genes were found to be involved in cell cycle progression, cell proliferation, and gene expression.

Both cross-species microRNA and mRNA gene expression microarrays were used by Maccani et al. in 2014 [32] to identify microRNA expression specific to high producing CHO cell lines and potential miRNA-mRNA interactions to understand the biological functions of the microRNAs. Human, mouse, and rat microRNAs were used to probe RNA extracts of five cell lines. These cell lines included high and low producing single-chain Fv-Fc fusion antibody cell lines, high and low producing Human Serum albumin cell lines, and a non-producing CHO cell line that are used to identify differentially expressed microRNAs. The 14 most significantly differentially expressed microRNAs were selected for qRT-PCR and 11, including mir-10b-5p, mir-21-5p, and mir-221-3p, were validated. A bioinformatics analysis was completed to identify biological functions of the microRNAs. Then, a CHO-K1 based mRNA microarray analysis was completed and potential microRNA-mRNA interactions were computed. For the 11 validated microRNAs, there were as few as zero negatively correlated differentially expressed targets, and as many as 46 [32].

A similar approach was used to profile the effects of mild hypothermia on HELA and CHO cells in a study by Emmerling et al. [33]. Microarrays of human microRNA probes for HELA cells expressing a recombinant adeno-associated virus (rAAV) were compared at two temperature conditions. For the CHO DG44 cells, the microarrays consisted of probes against mouse, rat, and human microRNAs. These microarrays were used to compare antibody expressing CHO cell lines at two temperature conditions. The investigators followed the microarrays with transient transfection of mir-483 mimics. It was determined that mir-483 regulates recombinant antibody and viral vector production in both CHO and Hela Cells, but is processed differently in the two species. Bioinformatics analysis identified potential targets, KANK4, PDK4, MAPK3, and CXCR4.

In 2016, Klanert et al. [34] used microarrays consisting of cross-species microRNA from human, mouse, rat, and viral microRNA to identify microRNAs that were associated with growth rate in several types of CHO cell lines expressing different recombinant products. They collected samples from cultures grown in different vessels, such as shaker flasks and bioreactors, in different media composition with and without serum, and in different growth phases, such as exponential and stationary, and analyzed the differential expression of microRNA by using microarrays. They identified 12 microRNAs, among them mir-222-3p, mir-23a-3p, and mir-29a-3p that appear to be associated with growth rate in multiple CHO cell lines.

### 2.3. microRNA Library Screen

**Figure 1:**
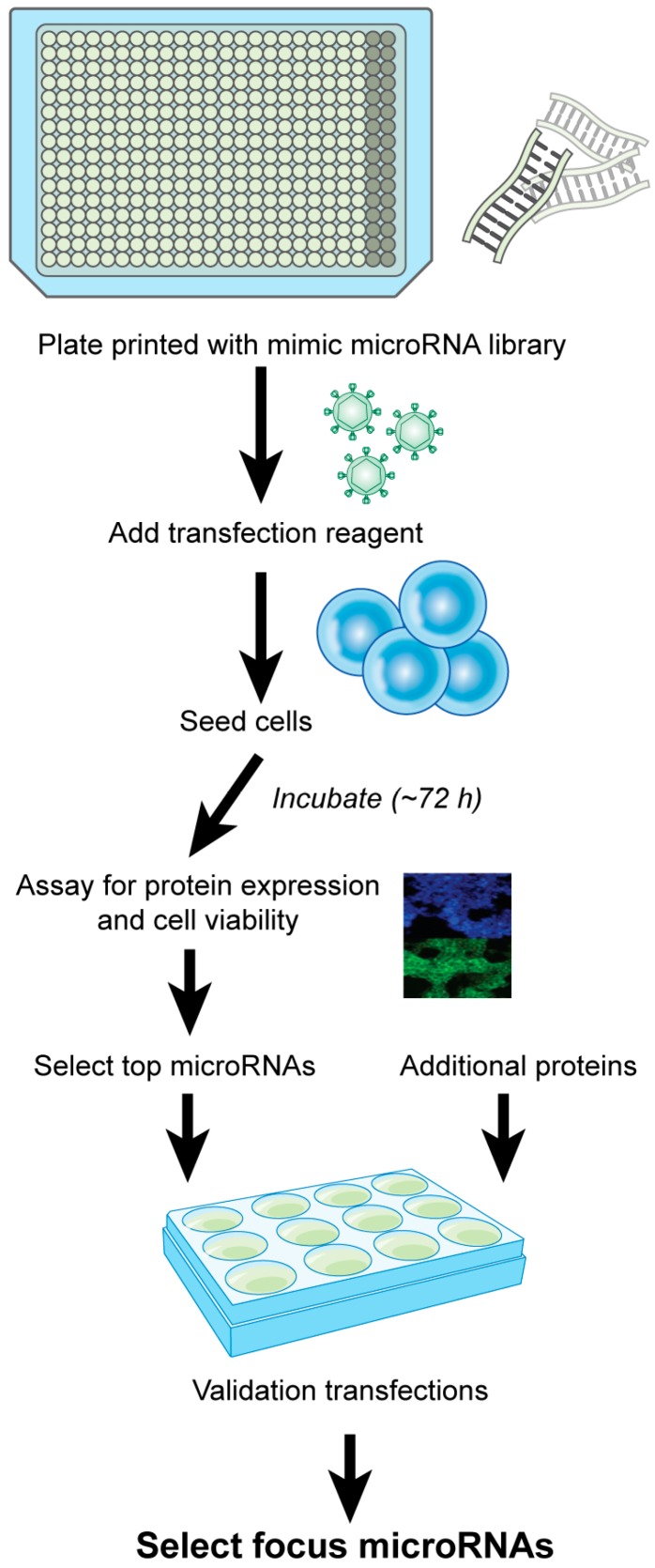
Example workflow for high-throughput microRNA library screen.

Another approach that is currently used for identifying specific microRNAs is screening microRNA mimic libraries. The screens are designed to identify microRNAs that improve specific cell properties, such as protein expression, viability, and growth. In this approach, instead of altering conditions and measuring different microRNA expression, the microRNA library is tested and microRNAs that showed the desired effect are selected for further evaluation. A sample workflow for a microRNA screen based on a study by Xiao et al. [37] is shown in Figure 1. In a 96-well plate format, a murine microRNA mimic library screen of 1139 microRNAs was used to determine microRNAs that improve the titer and specific productivity of SEAP producing CHO cell line [36]. After selecting the mir-30 family as a possible target for improving the SEAP productivity, stably over-expressing clones with members of the mir-30 family were generated [36]. In a follow up work, using bioinformatics and reporter assays, Fischer et al. [49] were able to identify members of the ubiquitin pathway as putative targets of the mir-30 family. The same high-content screen was later used to identify redundancy in microRNA control of cellular pathways [50]. The screen previously described, was used by Fischer et al. in 2015 [51] to identify mir-2861 as a potential target, confirm its expression in CHO cells, and evaluate its effect on recombinant protein expression in CHO cells. Using CHO cells expressing SEAP, they both transiently and stably transfected the cells with miR-2861 and siRNA against HDAC5, and analyzed apoptosis, cell cycle distribution, and productivity. Additionally, the link between mir-2861 and HDAC5 was examined. The screen was also used to identify mir-143 as an enhancer of productivity in CHO cells [52]. Schoellhorn et al. enhanced production by transiently and stably transfected SEAP and monoclonal antibody producing CHO cell lines with mir-143. Bioinformatics and qRT-PCR were used to identify that MAPK7 is affected by mir-143 and following this observation, they were able to improve specific productivity using a MAPK7 knockdown.

A high throughput human microRNA mimic screen in 96-well plate format was conducted by Strotbek et al. [35] using CHO cell line producing IgG. The initial screen that included 879 microRNAs was followed with a smaller scale validation screen composed of nine microRNAs to test the expression of recombinant human serum albumin from CHO cells. Based on the screening, stable CHO-IgG cell lines over-expressing microRNAs were constructed. Cell lines with over-expression of individual miR-557 or mir-1287 had no impact on productivity while a stable cell line over-expressing both miR-557 and mir-1287 had increased specific productivity and overall yield in a fed batch culture when compared with the parental cell line.

A later study by Fischer et al. [53], with the microRNA screen from Strotbek et al., used mir-557 to improve multiple antibody producing CHO cell lines including difficult to express proteins. The effect of mir-557 was tested by transient transfection in seven cell line conditions, including selection system (glutamine synthetase deficient and DHFR deficient), molecule type (IgG antibody, bispecific antibody, and bispecific antibody-scFv fusion), and expression level (high, medium, low, and very low). They then went on to generate stable miR-557 over-expressing CHO cell lines and used these for cell line development of easy to express and a difficult to express monoclonal antibody.

The microRNA screening approach was also used to determine microRNAs that improve the expression of neurotensin receptor in HEK 293 cells [37]. Following primary screen of 875 microRNA mimics in a 384-well plate format, 10 candidates were selected and validated with transfections in a 12-well plate format. The top candidates were tested for their effect on expression of two additional proteins for selecting microRNAs that were applicable for multiple protein types, of which mir-22-3p was selected for further study [37]. Recently, Meyer et al. [38] screened for microRNAs that increase antibody expression from transiently transfected HEK 293 cells by co-transfecting with plasmid containing the antibody with each of 875 microRNAs in the human microRNA library using a 384-well format. They found that adding valproic acid along with mir-337-5p or mir-26a-5p with transient transfection of the antibody improves the titer up to two-fold. They also showed that improved expression is protein dependent.

### 2.4. Next Generation Sequencing

Next generation sequencing (NGS) is an essential tool for “omics” studies, and, therefore, has often been implemented in noncoding RNA analysis [54]. In 2011, Hackl et al. [39] used NGS to sequence the small RNA transcriptome of six CHO cell lines. They identified and annotated sequence information for conserved and novel CHO microRNAs, creating tools for further microRNA research. From the list of microRNAs obtained, Jadhav et al. [40] tested the effect of over-expression of four microRNAs in CHO cells expressing recombinant erythropoietin-Fc fusion (EpoFc) by transient transfections of miRNA expression plasmids. They screened for growth and production characteristics, and selected mir-17 since it caused a 15.4% increase in growth rate and consequently increases final EpoFc titer. They also used qPCR to measure mRNA of known targets for mir-17, to show that the over-expression of the microRNA was enough to regulate the target genes. The work was followed by stable over-expression of miR-17 in a CHO cell line expressing EpoFc. The result was twofold increase in specific productivity and threefold increase in overall titer [55].

In 2014, Loh et al. [41] used NGS to profile microRNA in high and low expressing monoclonal antibody CHO cell lines. They identified a cluster of microRNAs that were differentially expressed in the high and low expressing cell lines and proceeded to individually and in combination express mir-17, mir-19b, mir-20a, and mir-92a. The highest clones showed 130–140% increase in specific productivity and titer and that mir-17, mir-19b, and mir-92a were correlated with increased protein expression. The study was followed later by bioinformatics and reporter assays to identify insig1 as the gene target of mir-92a in CHO cells [56].

By utilizing the observation that osmotic shifts in the media affect cell performance, Pfizenmaier et al. [42] studied mRNA and microRNA profile as a result of osmotic changes. After inducing an osmotic shift, they were able, by using NGS techniques, to identify mRNA and microRNAs that were differentially expressed at the different osmotic conditions, they followed by identifying targets that provided additional energy for recombinant protein biosynthesis. They identified several gene expression changes, but focused on microRNA changes that were related to cell cycle arrest and proliferation, selecting mir-183 for stable over expression, improving specific productivity.

In another study based on knowledge of productivity changes as a result of culture conditions, Stiefel et al. [43] used NGS to follow biphasic fed-batch cultivation, profiling low, high, and non-producing CHO cells, and investigating the effect of mild hypothermia. They identified 89 microRNAs that were differentially expressed between the different conditions. They then did a follow up validation experiment with 19 of these microRNAs transfecting them into CHO cells, measuring the effect on protein production, cell growth, apoptosis, and necrosis. The study wrapped up using Bioinformatics were used to identify target genes and relevant pathways that might be regulated.

## 3. Bioinformatics Methodologies

Interpretation of the experimental results obtained from any of the methods described in Section 2 for the identification of specific microRNAs, genes, and pathways cannot be done without specific bioinformatics tools. Web-databases and algorithms available for predicting mRNA targets of microRNA that have been used in the studies described in this review are summarized in Table 2. Additional detail for the basis and use of these algorithms can be found in numerous reviews and therefore will not be described here [57,58,59].

Identifying mRNA targets of identified microRNAs enables researchers to understand the pathways and mechanisms that are involved in improving recombinant protein expression [25,26,27,28,29,30,31,32,33,39,40,42,43,45,49,50,52,53,56]. In addition to detecting the mRNA-microRNAs interactions, investigators performed additional bioinformatics research to identify biological processes, gene ontology, and significant pathways that are affected by the targeted microRNAs. The researchers also aligned gene sequences between species, especially in the case of CHO cells where knowledge of genome is less evolved than that of the human genome [39,45,56]. These bioinformatics tools, also summarized in Table 2, help to provide a comprehensive analysis, ensuring a robust approach to improving recombinant protein production.

## 4. Additional Non-Coding RNA

Additional non-coding RNAs that were used to improve recombinant protein production include short hairpin RNA (shRNA), small interfering RNA (siRNA), mitochondrial genome-encoded small RNA (mitosiRNA), and sineUP. Other non-coding RNA molecules, such as PIWI-interacting RNA, and circular RNA, also have the potential to be used as targets for cellular engineering, but have yet to be tested [76].

### 4.1. Short Hairpin RNA

Short hairpin RNAs (shRNA) are DNA vector based RNA interference that are produced as single stranded molecules, 50–70 nucleotide stem-loop structures, and are cleaved by the nuclease Dicer to enter the RNA-induced silencing complex in the same way as siRNA, which triggers an RNAi response [77]. A study using an shRNA targeting dihydrofolate reductase (dhfr) showed improved productivity in CHO cells [78]. Based on the available information about the commonly used dhfr and Methotrexate (MTX) gene amplification system, Hong et al. designed an RNA silencing vector to target dhfr in dhfr deficient and wild type CHO cells with eGFP, to create a high producing cell line with improved stability without MTX. Wu et al. [79] followed up with enhancing IgG expression in CHO cells by targeting dhfr using the same RNA silencing vector.

### 4.2. Small Interfering RNA

Small interfering RNAs are double stranded 21–25 base pair RNAs that operate similarly to microRNAs regulating gene expression by degrading mRNA after transcription [80]. The major difference between siRNA and microRNA is that siRNA binds perfectly to a single gene, while microRNA imperfectly targets multiple genes [81]. Several studies were conducted using exogenous siRNA to target specific genes for improving protein expression [76,82,83,84,85]. These studies were primarily concentrated on targeting genes that are known to be involved with protein production, for example, genes that reduce apoptosis. Recently, a genome-wide siRNA screen was performed by Xiao et al. [86] in an analogous manner to the microRNA screens above. Transient transfections of siRNA for identifying gene targets that can affect protein expression were conducted in HEK 293 cells. By using large-scale high-throughput format, three siRNA for each gene were transfected into luciferase expressing HEK 293 cells, and their effect on luciferase production and cell viability was measured. The top 10 genes were confirmed with additional three siRNAs. From this study, OAZ1 was selected as a target gene for follow-up studies due to improvement expression of the luciferase protein in HEK293 cells [86].

### 4.3. Mitochondrial Genome-Encoded Small RNA

Mitochondrial genome-encoded small RNAs (mitosRNA) are a class of small RNAs that are derived in the mitochondria from ‘housekeeping’ non-coding RNAs and function similarly to microRNA [87]. In 2016, Pieper et al. [88] identified mitosRNA-1972 as a tool for improving the expression of IgG in CHO cells based on a BLAST alignment and knowledge about the function of the sequence. Once this was shown as a successful tool, they identified targets of the mitosRNA using next generation sequencing after transfecting with mitosRNA-1972, when comparing gene expression at multiple time points. ShRNA expression plasmid transfections were then used as follow-up studies to confirm Cers2 and Tbc1D20 as targets of mitosRNA-1978. These two genes were then used to co-engineer CHO-IgG producer cells with a combined knockdown with shRNA [88].

### 4.4. SINEUP RNA Levels

SINEUPs are a new class of natural and synthetic antisense long non-coding RNAs that require an invSINEB_2_ element whose effect is to upregulate translation of partially overlapping sense coding mRNAs with no consequence to RNA levels [89,90]. Patrucco et al. [91] manipulated these SINEUPs in CHO cells to improve secreted protein translation levels. SINEUPs that targeted cytosolic and secreted luciferase were also used to test the concept of SINEUPs and their ability to improve production. They then used SINEUPs to target therapeutic proteins, secreted ScFv, and a cytokine, successfully enhancing protein expression.

## 5. Summary and Conclusions

Small non-coding RNA particularly microRNA participate in many regulatory functions, including cell cycle regulation and proliferation. By implementing this information, it is possible to target specific, well-known pathways, to achieve improved performance of the cells. However, the available information on microRNA effect is limited and different approaches are needed to achieve improved cell function by using microRNAs. One of the approaches described in this review is through the identification of promising microRNA by utilizing microarrays.

Microarrays offer the ability to discover differentially regulated microRNAs based on conditions that are known to improve protein expression. Since the probes correspond to certain microRNAs or genes, the data analysis for microarrays, when compared with other technologies, such as Next Generation Sequencing, is relatively straight-forward, however, microRNA microarrays are limited to currently available microRNA probes. Cross-species microarrays have been used in place of CHO specific microarrays.

MicroRNA library screenings use cells treated with multiple microRNAs in small-scale high-throughput, format. To prepare for the screen, there is a need to optimize the transfections process and to choose an expressed protein that is possible to screen in this format, such as fluorescent marker. The microRNA library is growing with technology improvements, and the number of entries in miRbase database grew from 15,000 to almost 30,000 between 2010 and 2014 [68], and with it, the size of the screen. The data analysis of a microRNA screening is a bit more involved than for that of the microarray since there are cell counts, protein amounts, and specific protein production to consider each microRNA.

Next generation sequencing can be used in a similar manner as a microarray, but does not require specific microRNA probes. It is therefore easier to use for species that do not have fully developed tools such as CHO. RNA from a good producing condition is compared to that of the wild type and up or down regulated microRNAs are identified. Instead of probes that are attached to a chip, the RNA is transcribed to labelled cDNA libraries and then fully sequenced. This produces a significant amount of data that can be analyzed using multiple methods, each attaining slightly different results. Several review articles describe the differences between microarrays and next generation sequencing [92,93].

After identifying the microRNA target(s) from any of these screening technologies, validation is required. In the next step an improved producer stable cell line is created by over-expressing, or depleting the identified microRNA, in some cases, using multiple microRNAs together for a synergistic effect. Sometimes after investigating the mechanism, a gene knockdown or over expression is performed to further improve recombinant production. From the small non-coding RNA studies, numerous microRNAs were identified as potential targets for engineering high expressing cells.

An advantage of utilizing microRNA is the fact that a single construct targets multiple genes at the same time. However, this could also be a disadvantage since these targets are not fully elucidated. Some small ncRNA such as shRNA and siRNA are gene specific, narrowing the focus to one target gene and removing the uncertainty of undesired targets. As more information becomes available concerning small non-coding RNA molecules, more applications become possible for improving protein production, such as the use of mitosRNA and SINEUP. However, these agents are new and the technology has not yet evolved to give good screening tools to provide quick way to improve protein expression, but likely will be available in the future.

In summary: Using non-coding RNA as a method of modifying cell properties is an efficient alternative to classical cloning methods for improving recombinant protein expression since non-coding RNA does not require protein translation. Several methods are currently being applied for identifying and utilizing non-coding RNAs for improved recombinant protein expression from mammalian cells. By using approaches that consider known growth or production processes, and working backwards to identify the non-coding RNA that are related to that specific processes, or by conducting broad screening of microRNAs or siRNAs, specific targets have been identified. Because of this work, significant improvement in production level of several recombinant proteins have been achieved by affecting apoptosis, cell proliferation, and cell cycle distribution. Rapidly advancing technology continues to provide more methods for identifying and using different non-coding RNAs. Since the advancement in technology brings significant amount of data, there is a need for robust bioinformatics tools. As more information about non-coding RNAs and their mechanisms becomes available, their usefulness for improving recombinant protein expression from mammalian cells will continue to increase.

## Figures and Tables

**Table 1:** Summary of microRNA screening methodologies.

Year	Initial Screen	Researchers	Type of Cells	Conditions Evaluated in Initial Screen	Reference
**Previously identified microRNAs**					
2015	miR mimics and mir-34 sponge decoy	Kelly et al.	CHO	apoptosis and cell growth	[26]
2015	miR mimics and mir-23 sponge decoy	Kelly et al.	CHO	energy metabolism	[27]
**Microarray**					
2007	human, mouse and rat microRNA arrays	Gammell et al.	CHO	temperature shift	[25]
2009	human and mouse microRNA arrays	Koh et al.	HEK293	3 stages of batch culture	[28]
2011	human microRNA arrays	Barron et al.	CHO	temperature shift	[29]
2011	mouse and rat microRNA arrays	Druz et al.	CHO	apoptosis	[30]
2011	human, mouse and rat microRNA arrays	Lin et al.	CHO	producing lines compared to parental and MTX amplification	[31]
2014	cross-species microRNA and mRNA arrays	Maccani et al.	CHO	high producing cell lines compared to low producing cell lines	[32]
2016	human for HELA, mouse, rat and human for CHO microRNA arrays	Emmerling et al.	HELA and CHO	mild hypothermia	[33]
2016	human, mouse, rat, viral microRNAs	Klanert et al.	CHO	growth rate in multiple cell lines	[34]
**microRNA screen**					
2013	human microRNA library	Strotbek et al.	CHO	IgG	[35]
2014	murine microRNA library	Fischer et al.	CHO	SEAP	[36]
2015	human microRNA library	Xiao et al.	HEK293	neurotensin receptor	[37]
2017	human microRNA library	Meyer et al.	HEK293	antibody	[38]
**Next Generation Sequencing**					
2011	small RNA transcriptome	Hackl et al.	CHO	identified conserved and novel CHO microRNAs	[39]
2012	microRNA	Jadhav et al.	CHO	effects of overexpressing microRNA	[40]
2014	microRNA	Loh et al.	CHO	looking at profile of different expression level cultures	[41]
2016	microRNA and mRNA	Pfizenmaier et al.	CHO	osmotic shift	[42]
2016	microRNA	Stiefel et al.	CHO	biphasic fed batch cultivation of high low and non-producing CHO lines with mild hypothermia	[43]

**Table 2:** Summary of bioinformatics programs.

**microRNA Target Prediction**			
miRwalk	Collection of experimentally validated and predicted microRNA binding sites from multiple resources	http://mirwalk.uni-hd.de/	[60]
miRbase	Collection that provides a registry of published microRNA sequences	http://www.mirbase.org/	[61]
miRANDA algorithm	Algorithm that predicts microRNA targets based on sequence complementarity, energy binding and evolutionary conservation	http://www.microrna.org/	[62]
PITA	Database based on algorithms predicting targets based on site accessibility	https://genie.weizmann.ac.il/pubs/mir07/index.html	[63]
RNAhybrid	Database based on algorithms predicting targets based on minimum free energy hybridization	https://bibiserv.cebitec.uni-bielefeld.de/rnahybrid/	[64]
DIANA tools	Database based on algorithms predicting targets based on site recognition	http://diana.imis.athena-innovation.gr/DianaTools/index.php	[65]
targetScan	Database based on algorithms predicting targets based on site recognition	http://www.targetscan.org/vert_71/	[66]
EiMMo	Database based on algorithms predicting targets based on site recognition	http://www.clipz.unibas.ch//ElMMo3/index.php	[67]
miRtarbase	Database based on experimentally validated microRNA/mRNA interactions	http://mirtarbase.mbc.nctu.edu.tw/	[59]
mirdb	Database for microRNA target prediction and functional annotation	http://mirdb.org/	[68]
DAVID	Database for identifying gene ontology but can and has also been used for identifying microRNA targets	https://david.ncifcrf.gov/	[69]
**Biological Processes, Gene Ontology and Protein Identification**			
PANTHER	Database for gene ontology and gene clustering analysis and gene products	http://pantherdb.org/	[70]
MASCOT	Software program for identifying proteins	http://www.matrixscience.com/	
HomoloGene	Database containing information about genes that have been used to study homology between species as well as for providing information about gene function	https://www.ncbi.nlm.nih.gov/homologene	
GeneCards	Database containing information about genes that have been used to study homology between species as well as for providing information about gene function	http://www.genecards.org/	
BLAST	Basic local alignment search tool (i.e., Blast) utilizes the discontiguous megablast algorithm can be used to align gene sequences between species	https://blast.ncbi.nlm.nih.gov/Blast.cgi?CMD=Web&PAGE_TYPE=BlastHome	
edgeR	“R” software program package for differential expression analysis of RNA-seq data	https://bioconductor.org/packages/release/bioc/html/edgeR.html	[71]
maSigPro	“R” software program package for regression analysis and differential expression analysis of microarray and RNA-seq data	https://bioconductor.org/packages/release/bioc/html/maSigPro.html	[72]
LIMMA	“R” software program package for linear models and differential expression analysis of microarray data	https://bioconductor.org/packages/release/bioc/html/limma.html	[73]
Gorilla	Tool for identifying enriched gene ontology terms	http://cbl-gorilla.cs.technion.ac.il/	[74]
MGI Gene Ontology Term Finder	Gene ontology database primarily for mouse genes	http://www.informatics.jax.org/	[75]
Vmatch	Sequence analysis software	http://www.vmatch.de/	
MetaCore	Pathway and network analysis software	https://clarivate.com/products/metacore/	
Ingenuity Pathways Analysis	Pathway and network analysis software	https://www.qiagen.com/us/shop/analytics-software/biological-data-tools/ingenuity-pathway-analysis/	
